# Attitudes of Poles towards the COVID-19 Vaccine Booster Dose: An Online Survey in Poland

**DOI:** 10.3390/vaccines10010068

**Published:** 2022-01-02

**Authors:** Mateusz Babicki, Agnieszka Mastalerz-Migas

**Affiliations:** Department of Family Medicine, Wroclaw Medical University, 51-141 Wroclaw, Poland; agnieszka.mastalerz-migas@umed.wroc.pl

**Keywords:** COVID-19 vaccination, COVID-19 vaccine booster dose, attitudes towards vaccination

## Abstract

Introduction: COVID-19 vaccination has now become the most effective way to combat the pandemic, but there is a gradual decline in the protection that it offers over time. Therefore, the Food and Drug Administration (FDA) and EMA now recommend the use of the so-called booster dose, especially in at-risk groups. The purpose of the study was to assess the attitudes of Poles towards the recommendation to receive a booster dose of the COVID-19 vaccine and to evaluate the main reasons for refusing or delaying the decision. Material and methods: The study was based on a proprietary questionnaire distributed via the Internet. There were 1598 respondents, 54 of which did not consent to participate in the survey and/or did not complete the vaccination against SARS-CoV-2. As a result, 1528 surveys were included in the final analysis. The vast majority of the respondents, namely 1275 (83.4%), were female, and 772 (50.5%) were residents of cities with a population of over 250,000. Results: Out of all respondents, 38 (2.5%) had already received the COVID-19 vaccine booster dose and 1031 (67.4%) would like to receive it as soon as possible. Forty-five (2.9%) respondents reported that they were completely unwilling to take the booster dose. The occurrence of adverse events after primary vaccination were reported by 79.9% of the survey participants. The most common reasons why the respondents refused to be vaccinated are lack of confidence in the effectiveness of the booster dose and the occurrence of adverse events in them or their loved ones. Age, gender, residence, or relationship status were not shown to affect attitudes towards the expansion of the basic vaccination schedule. Conclusions: One in three respondents plans to delay or refrain from taking the COVID-19 vaccine booster dose. The main reason for refusal to be vaccinated is the belief that the previous vaccination provides sufficient protection.

## 1. Introduction

COVID-19, a disease caused by the SARS-CoV-2 virus, currently poses the greatest health challenge worldwide. Less than a few months after the outbreak of the pandemic, which was declared by the WHO on 10 March 2020, there were reports on the development of effective as well as safe vaccines against SARS-CoV-2, which were conditionally approved for widespread use at the end of 2020 [1,2,3,4,5,6]. After nearly a one-year follow-up, vaccination is known to dramatically reduce the risk of severe disease, hospitalisation and death from COVID-19 [7,8,9,10]. The level of immunity provided by it may vary depending on the vaccine used, the interval between doses as well as individual conditions and the virus variant [11,12,13,14]. Moreover, studies have shown that, over time, humoral immunity levels can decline and even eventually disappear [15,16]. Moreover, there is a group of patients, including those with reduced immune system function, in whom the basic schedule produced a low level of neutralising antibodies or the body did not produce them at all. Based on these data, studies were undertaken to investigate the administration of a supplemental dose in persons with impaired immunocompetence, which has been shown to significantly increase protection, and the administration of a booster dose in persons with a healthy immune system in order to extend the protection provided by primary vaccination [17,18]. Following reports of a gradual decline in protection after vaccination and the good effect of a booster dose six months after primary vaccination, many countries made the decision to administer booster doses [15,16,17,18]. The first country to implement booster vaccinations was Israel in June 2021. Other countries, including Germany, Russia, China, France and the United Arab Emirates, followed [19]. In September 2021, a booster dose was also made available in Poland, which was administered to more than 600,000 people within a month, representing approximately 30% of the eligible group at that time (minimum six months after primary vaccination) [20,21].

Despite the obvious benefits of vaccinations in Poland as well as in many countries around the world, many people do not plan to be vaccinated [22,23]. In Poland, over 19 million people or about 52% of the population have been vaccinated, which is far below the European Union average of 63% [24,25]. Despite active efforts to encourage vaccination, an increase in anti-vaccine attitudes has been observed. This phenomenon threatens the effectiveness of the vaccination programme in Poland and increases the risk of another pandemic wave. Moreover, it can also discourage people who have already been vaccinated from expanding the basic vaccination schedule. Undoubtedly, this phenomenon needs to be monitored in order to implement appropriate system solutions as soon as possible. With this in mind, the aim of this survey was to assess attitudes towards receiving a booster dose of the COVID-19 vaccination among fully vaccinated persons in Poland and to evaluate the main reasons for refusing to take the vaccine or delaying it.

To the authors’ knowledge, this is the first survey that assesses the attitudes of vaccinated individuals towards a potential extra vaccine dose.

## 2. Materials and Methods

### 2.1. Methodology

The survey was conducted using a proprietary questionnaire distributed through the social networking site Facebook.com. The place of distribution of the questionnaire included not only groups focusing on vaccination, COVID-19 vaccination or COVID-19 in Poland, but also general groups. The target group consisted of respondents over the age of 18, living in Poland, who had completed the basic COVID-19 vaccination schedule. The survey distribution period was 23 September 2021 to 3 October 2021. During this period, Europe was awaiting official recommendations for the use of a booster dose, which were published on 4 October 2021 [26]. At the time of data collection, in Poland it was allowed (from 1 September 2021) to administer an booster dose of vaccination in a group of patients with impaired immunocompetence, i.e., undergoing anticancer treatment or being treated with immunosuppressive agents after organ transplantation provided that they had previously completed a two-dose schedule using mRNA vaccines. During the course of the survey, the administration of the booster dose was also approved for individuals with normal immune systems, but only those over the age of 50 and for medical staff working in direct contact with patients, regardless of previous vaccine type [20,21].

Before participating in the survey, the respondents were informed about the nature and objectives of the survey. After that, informed consent was obtained from those willing to participate. The survey was approved by the Bioethics Committee of Wroclaw Medical University and was conducted in accordance with the Declaration of Helsinki.

The proprietary questionnaire consisted of both single-choice and multiple-choice questions. Before completing the questionnaire, the respondents were asked to consent to the survey and to indicate their vaccination status (no vaccination/incomplete vaccination (one dose of Comirnaty or Spikevax or AstraZeneca)/full vaccination (two doses of Comirnaty or Spikevax or AstraZeneca or 1 dose of Johnson & Johnson. If there was no consent and/or no vaccination or incomplete vaccination, the survey was automatically terminated. Once both criteria were met, the participant was redirected to the first section, which included questions defining their sociodemographic status such as age, gender, place of residence, education, and relationship status. The questionnaire also contained questions about whether the respondent was a medical professional and which vaccine he or she received (Pfizer/Moderna/AstraZeneca/Johnson & Johnson/mixed schedule), and if there were any adverse events after vaccination. The presence and type of chronic conditions were also assessed. Subsequently, attitudes towards receiving the booster dose were assessed by means of the following question: “Do you plan to take a booster dose of the COVID-19 vaccine?”, with the following possible responses: “I am already vaccinated/Yes, as soon as possible/Yes, in a few months (up to a year)/Yes, but after a year/I cannot decide/No, but I might consider it in the future/No, never”. In the case of responses other than “I am already vaccinated” or “Yes, as soon as possible”, the respondents were asked why they did not plan to be vaccinated/planned to delay taking the booster dose.

For greater methodological clarity, an English-language version of the questionnaire is included as supplementary materials.

### 2.2. Statistical Analysis

Variables were qualitative and quantitative. Basic descriptive statistics were used. The chi-square test was used to determine the relationship between the compared ordinal variables, and the value of Cramer’s V coefficient was additionally assessed. In addition, Pearson correlation assessment was performed, evaluating the degree of dependence between quantitative variables.

A statistical significance level of *p* < 0.05 was assumed in all tests. The analysis was conducted with the use of Statistica 13.0, StatSoft (Hamburg, Germany).

## 3. Results

### 3.1. Description of the Study Group

There were 1582 respondents, 54 of which did not consent to participate in the survey and/or did not complete the vaccination schedule. As a result, 1528 surveys were included in the final analysis. A detailed description of the study group is presented in Table 1.

The vast majority of the respondents were women 1275 (83.4%) and people with higher education (77.8%). Five hundred and fifty-seven respondents (36.5%) were healthcare workers and 595 respondents (38.9%) had at least one chronic condition. The most common vaccine administered to the respondents was Pfizer/BioNTech’s Comirnaty vaccine (74.1%), while the schedule of 37 (2.4%) respondents was mixed and used mRNA vaccine and a vector vaccine. 79.9% of the respondents referred to the occurrence of side effects after the basic schedule, with most of them being mild—1006 (65.8%).

### 3.2. Social Attitudes towards the COVID-19 Vaccine Booster Dose

Out of all respondents, 38 (2.5%) had already received the COVID-19 vaccine booster dose and 1031 (67.4%) would like to receive it as soon as possible. Only 45 (2.9%) respondents report that they are completely unwilling to take the booster dose. For those refusing to take/delaying the booster dose, the most common reason is the belief that there is a high level of protection after full vaccination. Fifty-three respondents (11.3%) believe that the so-called “hybrid” immunity (having recovered from COVID-19 and completed the vaccination schedule) provides adequate protection without the need to take the booster dose. 15.4% of the respondents were concerned that the booster dose might cause complications in the future. More than one reason is indicated by 24.6% of the respondents. A detailed summary of attitudes towards the booster dose and reasons for refusal/delay are given in Table 2.

### 3.3. Effects of Sociodemographic Variables and Concerns on the Willingness to Take the Booster Dose

The analysis of the impact of sociodemographic variables showed that the willingness to get the vaccine booster dose increases with age (r = 0.05; *p* = 0.039). In contrast, gender, place of residence, level of education, and relationship status do not affect attitudes towards vaccination. Medical professionals have been shown to be more willing to be vaccinated, with 74.3% saying they have already been vaccinated or want to get the vaccine as soon as possible. When chronic conditions coexist, the willingness to receive a booster dose of the COVID-19 vaccine increases (*p* < 0.001), but as the number of chronic conditions increased, the willingness to be vaccinated does not increase significantly (r = 0.031; *p* = 0.226). Of those who do not believe in the effectiveness of the booster dose, as many as 37.9% never intend to take it and 43.2% may consider it in the future (*p* < 0.001). In contrast, 40.8% of the respondents who believe their level of protection after primary vaccination is high enough and 45.3% of those who believe hybrid immunity is sufficient intend to get the booster dose within the next 12 months. Table 3 presents a detailed comparative summary of the univariate analysis.

## 4. Discussion

The results of this study point to moderate acceptance of getting a booster dose of the COVID-19 vaccine. Among 1528 individuals, 1031 (67.4%) want to get their booster dose as soon as possible and 38 (2.5%) have already received the booster dose. Forty-five (2.9%) respondents do not intend to get vaccinated. 79.9% of the respondents report that they experienced adverse events of varying degrees of severity after full vaccination. The presence and severity of these symptoms have been shown to affect the decision regarding the booster dose. This appears to be consistent with previous reports, which state that the likelihood of getting vaccinated decreases with the increase in adverse events [22,27]. In this study, the presence of adverse events after full vaccination was indicated as one of the most common reasons for refusal to take the booster dose. Presumably, this may be due to the concern that the booster dose may generate more severe adverse effects than basic vaccine doses. At this time, there is a lack of studies describing adverse events after booster doses. A Pfizer clinical trial covering 306 participants aged 18–60 showed that adverse events were similar to those occurring after the basic full vaccination. Local and systemic symptoms of mild to moderate severity were predominant [28,29]. Patient observations from the v-safe database of 21,191 subjects showed slightly more frequent local reactions and less frequent systemic reactions. However, registration in the database by the patient is voluntary, which is a methodological limitation [29,30]. The occurrence of adverse events after the booster dose is the issue that requires ongoing observation to better understand the potential effects of taking the dose. In the past, a decline in confidence in COVID-19 vaccines has been observed following the emergence of information on both an increased risk of thrombosis and myocarditis observed as vaccination programmes progressed in many countries around the world [22,31,32,33].

Another reason given by those refusing to take the booster dose/delaying it is the lack of faith in its effectiveness or the belief that the basic vaccination schedule ensures an adequate and long-lasting level of protection. As already known from several observations, both the level of the immune response after taking the booster dose and its duration vary and decrease with time [15,16,34]. However, we still do not know the level of antibodies or any other immunological marker that is measurable and correlated with high vaccine dose efficacy [19]. In addition, some of those vaccinated have not developed immunity or have low levels of immunity despite completing the basic schedule [17,18]. This was most common in patients with impaired immune function, who were the first people recommended to receive the booster dose. Current studies show that the use of the booster dose in this group of patients induces or enhances and prolongs the level of protection while reducing the risk of severe COVID-19 [17,18]. Data on patients with normal immune systems are scarce. One such study is the observation made by E. Saight et al., where among 346 medical professionals who received the booster dose, 95.7% experienced an increase in the IgG antibody titer from 3.67 (IQR 2.00–7.10) to >150 (upper limit of quantification) measured 10 days after administration [35]. Further, a large study carried out in Israel showed that the booster dose reduced both the risk of SARS-CoV-2 infection and its severity nearly 19.5-fold [36]. Thus, those results indicate the high efficacy of the booster dose. In addition, it is also important to remember about the cellular response that is produced by vaccinations in the form of SARS-CoV-2-specific CD4+ and CD8+ T cells that are involved in fighting the disease, and that the booster dose causes an increase in their titer [37,38,39].

The authors are aware of the strengths and weaknesses of the study. An undisputed strength of the study is its innovative nature, as to the authors’ previous knowledge, attitudes of vaccinated individuals towards the expansion of the basic vaccination schedule have not been investigated. On the other hand, a limitation of the study is the data collection methodology. Online distribution has many limitations. Firstly, the authors are unable to determine the response rate and the total number of people reached by the survey. Secondly, online surveys run the risk of producing a result depending on the independent selection of the study group. To avoid this, the authors distributed the questionnaire not only among groups related to vaccinations and COVID-19 in Poland but also among general groups. It should also be borne in mind that the surveyed group is not representative of Polish society. The overwhelming prevalence of women, people in relationships, individuals with higher education and those living in large cities may have affected the final results.

In summary, the willingness to get the vaccine booster dose in Poland is low. As many as one in three respondents plan to delay taking the booster dose or refrain from taking it. This issue arouses a lot of emotions and controversy not only among non-medical people but also among specialists, whose opinions sometimes differ. According to epidemiologists, current data are not convincing enough to implement general booster dose administration. It is estimated that from a public health perspective, it will be far more beneficial to vaccinate more people than to increase immunity in vaccinated individuals [40]. The rationale for administering vaccine booster doses while billions of people worldwide are still waiting to be vaccinated should be considered. For example, in Africa, only 5.1% of people are vaccinated [25]. Moreover, we do not know whether the booster dose will significantly prolong COVID-19 protection duration or whether it will only provide short-term protection [40]. On the other hand, immunity improvement may help reduce virus transmission. According to studies, SARS-CoV-2 virus levels are approximately 40% lower in vaccinated individuals. This may help to break the chain of transmission, thus protecting those who, for various reasons, cannot or do not wish to get vaccinated, including children [41].

In the light of the low percentage of vaccinated persons in Poland, the gradual decrease in the level of protection among vaccinated persons, as well as the risk of development of further SARS-CoV-2 variants, it is necessary to constantly monitor social attitudes towards vaccination because, as we know, effective vaccination will not be possible without social acceptance [42].

## 5. Conclusions

The acceptance of booster doses in Poland is quite low. One-third of the respondents plan to delay booster vaccination or refrain from taking the booster dose. The main reason for refusing to take or delaying the vaccine booster dose is the conviction that there is a sufficient level of protection after primary vaccination and after having recovered from COVID-19. The occurrence and severity of adverse events after the basic vaccination schedule affects attitudes towards the booster dose. In view of the observed public reluctance to vaccination, it is necessary to continuously monitor attitudes towards both primary vaccination and booster doses as well as to actively encourage the public to get vaccinated.

## Figures and Tables

**Table 1:** Characteristics of the study group.

Variable		N (%)/M ± SD
Age		34 ± 10.31
Sex	Female	1275 (83.4%)
	Male	253 (16.6%)
Place of residence	Rural area	241 15.8%
	City of up to 50,000 inhabitants	232 (15.2%)
	City of 50,000–250,000 inhabitants	283 (18.5%)
	City of more than 250,000 inhabitants	772 (50.5%)
Level of education	Primary	8 (0.5%)
	Lower secondary	6 (0.4%)
	Vocational	16 (1%)
	Secondary	309 (20.2%)
	Higher (university degree)	1189 (77.8%)
Relationship status	Single	288 (18.8%)
	Partnership	374 (24.5%)
	Married	866 (56.7%)
Healthcare professional	No	971 (63.5%)
	Yes	557 (36.5%)
Chronic conditions	No	933 (61.1%)
	Yes	595 (38.9%)
	Cardiovascular system, yes	120 (7.9%)
	Respiratory system, yes	93 (6.1%)
	Nervous system, yes	26 (1.7%)
	Oncological, yes	12 (0.8%)
	Psychiatric, yes	121 (7.9%)
	Dermatological, yes	59 (3.9%)
	Endocrinological, yes	303 (19.8%)
	Other than listed, yes	105 (6.9%)
Vaccination history	No	33 (2.2%)
	Only mandatory	761 (47.6%)
	Mandatory and recommended	767 (50.2%)
COVID-19 vaccines taken	Pfizer/BioNTech	1133 (74.1%)
	Moderna	154 (10.1%)
	AstraZeneca	200 (13.1%)
	Johnson&Johnson	4 (0.3%)
	Mixed	37 (2.4%)
Adverse events following vaccination	None	307 (20.1%)
	Mild	1006 (65.8%)
	Moderate	210 (13.7%)
	Severe	5 (0.4%)

**Table 2:** Willingness to take the vaccine booster dose and reasons for refusing to be vaccinated/delaying the booster dose.

Variable		N (%)
Willingness to take the booster dose	No, never	45 (2.9%)
	No, but maybe in the future	118 (7.7%)
	I cannot decide	103 (6.7%)
	Yes, but in a year or more	17 (1.1%)
	Yes, within a year	176 (11.5%)
	Yes, as soon as possible	1031 (67.4%)
	I am already vaccinated	38 (2.5%)
Reason for refusing to take the booster dose/delaying the booster dose (n = 467)	Lack of faith in the effectiveness of the booster dose	37 (7.9%)
	Severe adverse event after taking the vaccine (hospitalisation)	9 (1.9%)
	Severe adverse event after taking the vaccine (without hospitalisation)	19 (4.0%)
	Moderate adverse event	58 (12.4%)
	The vaccine doses that I have already received give me a sufficient sense of immunity	211 (45.2%)
	Severe adverse event in family/friends	13 (2.7%)
	Logistical difficulties e.g., transportation	7 (1.5%)
	Concern about future complications	72 (15.4%)
	I am a recovered patient and I think that the recovery gives me the right protection	53 (11.3%)
	Other reasons	139 (29.7%)

**Table 3:** Willingness to take the vaccine booster dose in relation to sociodemographic variables, vaccination history, chronic conditions and the vaccine used in the basic vaccination schedule.

Variable		Willingness to Be Vaccinated N (%)							Cramér’s V	*p*
		No, Never	No, But Maybe in the Future	I Cannot Decide	Yes, But in a Year or More	Yes, within a Year	Yes, as Soon as Possible	I Am Already Vaccinated		
Sex	Female	36 (2.8)	102 (8.0)	92 (7.2)	12 (0.9)	149 (11.7)	855 (67.1)	29 (2.3)	0.071	0.282
	Male	9 (3.6)	16 (6.3)	11 (4.3)	5 (1.9)	27 (10.7)	176 (69.6)	9 (3.6)		
Place of residence	Rural area	9 (3.7)	25 (10.3)	18 (7.5)	4 (1.7)	24 (9.9)	157 (65.2)	4 (1.7)	0.068	0.284
	City of up to 50,000 inhabitants	7 (3.0)	23 (9.8)	18 (7.8)	2 (0.9)	25 (10.8)	152 (65.5)	5 (2.2)		
	City of 50,000–250,000 inhabitants	10 (3.5)	17 (6.0)	19 (6.7)	3 (1.1)	48 (16.9)	178 (62.9)	8 (2.8)		
	City of more than 250,000 inhabitants	19 (2.5)	53 (6.9)	48 (6.2)	8 (1.0)	79 (10.2)	544 (70.5)	21 (2.7)		
Level of education	Primary	0	1 (12.5)	2 (25.0)	0	2 (25.0)	3 (37.5)	0	0.691	0.123
	Lower secondary	1 (16.7)	0	0	0	1 (16.7)	4 (66.6)	0		
	Vocational	0	0	2 (12.5)	0	3 (18.8)	11 (68.7)	0		
	Secondary	14 (4.5)	26 (8.4)	17 (5.5)	2 (0.7)	33 (10.7)	216 (69.9)	1 (0.3)		
	Higher (university degree)	30 (2.5)	91 (7.7)	82 (6.9)	15 (1.3)	137 (11.5)	797 (67.0)	37 (3.1)		
Relationship status	Single	4 (1.6)	25 (8.7)	18 (6.3)	7 (2.4)	28 (9.7)	198 (68.8)	8 (2.8)	0.080	0.087
	Partnership	9 (2.4)	23 (6.2)	27 (7.2)	3 (0.8)	39 (10.4)	258 (69.0)	15 (4.0)		
	Married	32 (3.7)	70 (8.1)	58 (6.7)	7 (0.8)	109 (12.6)	575 (66.4)	15 (1.7)		
Healthcare professional	No	32 (3.3)	83 (8.6)	75 (7.7)	13 (1.3)	113 (11.6)	651 (67.0)	4 (0.4)	0.189	<0.001
	Yes	13 (2.4)	35 (6.3)	28 (5.0)	4 (0.7)	63 (11.3)	380 (68.2)	34 (6.1)		
Chronic conditions	No	32 (3.4)	74 (7.9)	65 (7.0)	14 (1.5)	123 (13.2)	600 (64.3)	25 (2.7)	0.099	0.014
	Yes	13 (2.2)	44 (7.4)	38 (6.4)	3 (0.5)	53 (8.9)	431 (72.4)	13 (2.2)		
	Cardiovascular system	5 (4.2)	4 (3.3)	6 (5.0)	2 (1.7)	10 (8.3)	90 (75.0)	3 (2.5)	0.068	0.244
	Respiratory system	2 (2.2)	9 (9.7)	6 (6.5)	0	8 (8.6)	65 (69.9)	3 (3.21	0.045	0.687
	Nervous system	1 (3.85)	2 (7.7)	2 (7.7)	0	2 (7.7)	17 (65.4)	2 (7.7)	0.048	0.816
	Oncological	1 (8.3)	3 (25.0)	0	0	0	8 (66.7)	0	0.075	0.173
	Psychiatric	2 (1.7)	10 (8.3)	9 (7.4)	0	14 (11.6)	86 (71.1)	0	0.062	0.111
	Dermatological	0	4 (6.8)	4 (6.8)	0	5 (8.4)	43 (72.9)	3 (5.1)	0.057	0.315
	Endocrinological	9 (3.0)	23 (7.5)	21 (6.9)	2 (0.7)	23 (7.6)	222 (73.3)	3 (1.0)	0.085	0.052
	Other	3 (2.9)	9 (8.6)	4 (3.8)	0	13 (12.4)	72 (68.6)	4 (3.8)	0.049	0.544
Vaccination history	No	2 (6.1)	3 (9.1)	4 (12.0)	0	5 (15.2)	18 (54.6)	1 (3.0)	0.201	<0.001
	Only mandatory	34 (4.7)	92 (12.6)	70 (9.6)	12 (1.7)	99 (13.6)	413 (56.7)	8 (1.1)		
	Mandatory and recommended	9 (1.2)	23 (3.0)	29 (3.8)	5 (0.7)	72 (9.3)	600 (78.2)	29 (3.8)		
COVID-19 vaccines taken	Pfizer/BioNTech	30 (2.7)	85 (7.5)	78 (6.9)	12 (1.1)	133 (11.7)	760 (67.2)	35 (3.1)	0.138	<0.001
	Moderna	1 (0.7)	17 (11.0)	12 (7.8)	2 (1.3)	22 (14.3)	99 (64.3)	1 (0.7)		
	AstraZeneca	4 (2.0)	7 (3.5)	9 (4.5)	2 (1.0)	16 (8.0)	160 (80.0)	2 (1.0)		
	Johnson&Johnson	1 (25.0)	0	1 (25.0)	0	1 (25.0)	1 (25.0)	0		
	Mixed	9 (24.3)	9 (24.3)	3 (8.2)	1 (2.7)	4 (10.8)	11 (29.7)	0		
Adverse events following vaccination	None	8 (2.6)	23 (7.5)	22 (7.2)	1 (0.3)	27 (8.8)	220 (71.7)	6 (1.9)	0.142	<0.001
	Mild	15 (1.5)	65 (6.5)	64 (6.4)	15 (1.5)	123 (12.2)	696 (69.2)	28 (2.8)		
	Moderate	22 (10.5)	27 (12.9)	17 (8.1)	1 (0.5)	25 (11.9)	114 (54.2)	4 (1.9)		
	Severe	0	3 (60.0)	0	0	1 (20.0)	1 (20.0)	0		
Reason for refusing to take the booster dose/delaying the booster dose	Ineffectiveness of the booster dose	14 (37.9)	16 (43.2)	7 (18.9)	0	0	-	-	0.343	<0.001
	Severe adverse event after primary vaccination (hospitalisation)	2 (22.2)	5 (55.6)	1 (11.1)	0	1 (11.1)	-	-	0.126	0.299
	Severe adverse event after primary vaccination (without hospitalisation)	7 (36.8)	6 (31.6)	3 (15.8)	0	3 (15.8)	-	-	0.202	0.006
	Moderate adverse event	9 (15.5)	16 (27.6)	13 (22.4)	13 (5.2)	17 (29.3)	-	-	0.094	0.421
	Appropriate level of protection after basic vaccination schedule	13 (6.2)	61 (28.9)	39 (18.5)	12 (5.7)	86 (40.8)	-	-	0.176	0.005
	Severe adverse event in friends/family	8 (61.5)	3 (23.1)	2 (15.4)	0	0	-	-	0.304	<0.001
	Logistical difficulties	0	2 (28.6)	1 (14.3)	0	4 (57.1)	-	-	0.063	0.602
	Fear of future complications	12 (16.7)	31 (43.0)	19 (26.4)	0	10 (13.9)	-	-	0.260	<0.001
	Adequate protection after having recovered from COVID-19	6 (11.3)	13 (24.5)	8 (15.1)	2 (3.8)	24 (45.3)	-	-	0.071	0.646
	Other than listed	11 (7.9)	26 (18.7)	33 (23.7)	4 (2.9)	65 (46.8)	-	-	0.137	0.064

## Data Availability

The data presented in this study are available on request from the corresponding author.

## Supplementary Materials

The following are available online at https://www.mdpi.com/article/10.3390/vaccines10010068/s1, Survey.
