# Supplementary material for: Attitudes of Poles towards the COVID-19 Vaccine Booster Dose: An Online Survey in Poland
Source: Vaccines (Basel). 2022 Jan 2;10(1):68. doi: 10.3390/vaccines10010068 (PMC8778409; doi:10.3390/vaccines10010068)
Supplement: Supplementary file 1 [file vaccines-10-00068-s001.zip › vaccines-1520737-supplementary.pdf]

Questionnaire

Ladies and Gentlemen,

As employees of the Department of Family Medicine at the Wroclaw Medical University, we would like to invite you to participate in a survey aimed at assessing the attitudes of Poles towards the booster/supplemental dose of COVID-19 vaccination. The survey is fully anonymous, voluntary and addressed to all individuals who have completed the full COVID-19 vaccination schedule.

You may withdraw from the survey at any stage without giving any reason.

1. Would you like to take part in the survey? Yes/No
2. Have you been vaccinated against COVID-19 Yes, full cycle (2 doses of Pfizer/Moderna/AstraZeneca or 1 dose of Johnson&Johnson) / Yes, but I have not completed the basic vaccination schedule / No
3. Sex: Female [ ] Male [ ]
4. Age: ..... years of age
5. Place of residence:
   a) Rural area
   b) City <50,000 residents
   c) City of 50,000–250,000 residents
   d) City >250,000 residents
6. Education:
   a) Primary
   b) Lower secondary
   c) Vocational
   d) Secondary
   e) Higher (university degree)
7. What is your marital status?:
   a) I am not in a relationship/ I am divorced
   b) Civil partnership/ Informal relationship
   c) Married
8. Are you a healthcare professional?
   a) Yes
   b) No
9. If yes, what is your profession?
   a) Medical doctor
   b) Dentist
   c) Nurse and midwife
   d) Paramedic
   e) Laboratory diagnostician

f) Technician
g) Pharmacist
h) Other

10. Do you suffer from any chronic diseases?
a) Yes
b) No

11. If yes, what kind? (you can choose more than one option)
a) Cardiovascular diseases (e.g. hypertension, cardiac failure)
b) Respiratory diseases (e.g. asthma, COPD)
c) Neurological diseases (e.g. MS, epilepsy)
d) Oncological diseases (e.g. breast cancer, colorectal cancer, etc.).
e) Mental illnesses (e.g. depression, anxiety disorders, addictions)
f) Skin diseases (e.g. psoriasis, AD)
g) Endocrine disorders (e.g. thyroid, thyroid gland, diabetes)
h) Other

12. What preparation have you been vaccinated with against COVID-19?
a) Pfizer BioNtech
b) Moderna
c) AstraZeneca
d) Johnson & Johnson
e) Mixed schedule

13. Have you had any adverse reactions after the COVID-19 vaccination?
a) Yes, serious (requiring hospitalisation)
b) Yes, moderate (e.g. dyspnoea, temperature above 38.5 degrees Celsius, malaise – symptoms lasting more than 72 hours)
c) Yes, mild (e.g. low-grade fever, pain/swelling at the injection site)
d) No

14. Have you ever received any mandatory and/or recommended vaccines?
a) Yes, only mandatory
b) Yes, mandatory and recommended
c) No

14. Are you planning to get vaccinated against COVID-19?
a) I have already received the booster/supplemental dose
b) Yes, as soon as possible
c) Yes, but in a few months (up to a year)
d) Yes, but in a year or more
e) I cannot decide
f) No, but I might consider it in the future
g) No, never

16. Why do you want to wait to take the booster dose/why do you not want to take the booster dose? (multiple choice question)

a) I don't believe in the effectiveness of the booster dose
b) I had severe adverse effects after vaccination (requiring hospitalisation or medical advice) and I fear it will be worse after the next dose
c) I had severe adverse effects after vaccination (requiring outpatient medical advice) and I fear it will be worse after the next dose
d) I had moderate adverse effects after vaccination (not requiring hospitalisation or medical advice) and I fear it will be worse after the next dose
e) I believe that the current schedule gives me a sufficient level of protection
f) A friend/family member had severe adverse effects after vaccination and I am afraid of experiencing them myself
g) Logistical difficulties (e.g. transport to the vaccination facility, date of vaccination)
h) I am concerned about possible complications in the future
i) I'm a recovered patient and I think it gives me sufficient protection
j) Other
